# Analysis of the infant gut microbiome reveals metabolic functional roles associated with healthy infants and infants with atopic dermatitis using metaproteomics

**DOI:** 10.7717/peerj.9988

**Published:** 2020-09-25

**Authors:** Amornthep Kingkaw, Massalin Nakphaichit, Narissara Suratannon, Sunee Nitisinprasert, Chantha Wongoutong, Pantipa Chatchatee, Sucheewin Krobthong, Sawanya Charoenlappanit, Sittiruk Roytrakul, Wanwipa Vongsangnak

**Affiliations:** 1 Department of Zoology, Faculty of Science, Kasetsart University, Bangkok, Thailand; 2 Department of Biotechnology, Faculty of Agro-Industry, Kasetsart University, Bangkok, Thailand; 3 Pediatric Allergy & Clinical Immunology Research Unit, Division of Allergy and Immunology, Department of Pediatrics, Faculty of Medicine, Chulalongkorn University, King Chulalongkorn Memorial Hospital, The Thai Red Cross Society, Bangkok, Thailand; 4 Department of Statistics, Faculty of Science, Kasetsart University, Bangkok, Thailand; 5 Proteomics Research Laboratory, National Center for Genetic Engineering and Biotechnology, National Science and Technology Development Agency, Pathum Thani, Thailand; 6 Functional Ingredients and Food Innovation Research Group, National Center for Genetic Engineering and Biotechnology, National Science and Technology Development Agency, Pathum Thani, Thailand; 7 Omics Center for Agriculture, Bioresources, Food, and Health, Kasetsart University (OmiKU), Bangkok, Thailand

**Keywords:** Atopic dermatitis, Gut microbiome, Infants, Metabolic function, Metaproteomics, Bioinformatics

## Abstract

The infant gut microbiome consists of a complex and diverse microbial community. Comprehensive taxonomic and metabolic functional knowledge about microbial communities supports medical and biological applications, such as fecal diagnostics. Among the omics approaches available for the investigation of microbial communities, metaproteomics-based analysis is a very powerful approach; under this method, the activity of microbial communities is explored by investigating protein expression within a sample. Through use of metaproteomics, this study aimed to investigate the microbial community composition of the infant gut to identify different key proteins playing metabolic functional roles in the microbiome of healthy infants and infants with atopic dermatitis in a Thai population-based birth cohort. Here, 18 fecal samples were analyzed by liquid chromatography-tandem mass spectrometry to conduct taxonomic, functional, and pathway-based protein annotation. Accordingly, 49,973 annotated proteins out of 68,232 total proteins were investigated in gut microbiome samples and compared between the healthy and atopic dermatitis groups. Through differentially expressed proteins (DEPs) analysis, 130 significant DEPs were identified between the healthy and atopic dermatitis groups. Among these DEPs, eight significant proteins were uniquely expressed in the atopic dermatitis group. For instance, triosephosphate isomerase (TPI) in *Bifidobacteriaceae* in the genus* Alloscardovia* and demethylmenaquinone methyltransferase (DMM) in *Bacteroides* were shown to potentially play metabolic functional roles related to disease. PPI network analysis revealed seven reporter proteins showing metabolic alterations between the healthy and disease groups associated with the biosynthesis of ubiquinone and other quinones as well as the energy supply. This study serves as a scaffold for microbial community-wide metabolic functional studies of the infant gut microbiome in relation to allergic disease.

## Introduction

The microbiome plays an essential role in human immune maturation by mediating host immune responses, starting from the first month of life (*Cianci et al., 2018*). Simultaneously, host and environmental factors can influence microbial colonization and functions. The establishment of the microbiome begins at birth, after which the microbiome increases in diversity and stability over the first three years of life (*Fouhy et al., 2012; Lozupone et al., 2012*). Several bacterial taxa, such as *Bifidobacterium* and *Bacteroides*, predominantly colonize healthy infants who are born via vaginal delivery or breastfed (*Jost et al., 2012*), while in babies born via *cesarean* section, a different bacterial composition is established (*Azad et al., 2013; Dominguez-Bello et al., 2010*). Gut microbial dysbiosis is known to be associated with chronic inflammatory disorders, such as allergy (*Zhao, Ho & Bunyavanich, 2019*), inflammatory bowel disease (*Macfarlane et al., 2009*), and cancer (*Gopalakrishnan et al., 2018*).

Atopic dermatitis is the most common chronic inflammatory skin condition that usually starts in early childhood. It is the first allergic manifestation of a process known as the atopic march, a progression from atopic dermatitis to other allergic diseases, such as allergic rhinitis and asthma. The complex pathophysiology of atopic dermatitis includes immune dysregulation and skin barrier defects. Because signaling molecules produced by the microbiome can shape host mucosal and systemic immune responses, gut dysbiosis is expected to play important roles in the mechanisms underlying atopic dermatitis (*Sugita & Akdis, 2020; Tanaka & Nakayama, 2017*). Both genetic and environmental factors modulate gut microbiome colonization and function and influence the risk of the development of atopic dermatitis. Considering that genetics, dietary habits and living environments in Southeast Asia are different from those in other regions of the world, studies from Western countries are not directly applicable to Thai population.

Gut microbiomics often involves a 16S rRNA gene sequencing approach to provide information about gut microbial diversity. However, this approach critically depends on the optimization of the DNA extraction method and the choice of primers for amplifying the specific regions of 16S rRNA genes prior to sequence analysis (*Walker et al., 2015*). Subsequent whole-genome shotgun sequencing has been a key driving force in providing information about total gut microbial diversity. Nevertheless, this method is costly and time-consuming in terms of genome assembly, annotation and analysis of the microbiome. To overcome this challenge, metaproteomics has recently emerged as an alternative approach; metaproteomics can identify and quantify proteins from microbial communities at a large scale and thus provide direct insight into these microbial communities at the molecular level (*Kleiner, 2019*). Recently, there have been a number of studies on metaproteomic approaches for investigating the infant gut microbiome to identify taxonomic differences associated with several factors, such as age, sex, the mode of delivery and different treatments (*Cortes et al., 2019*). Moreover, metaproteomic studies have been used to investigate microbial communities associated with human diseases, such as inflammatory bowel disease (*Zhang et al., 2018*), chronic kidney disease (*Zybailov et al., 2019*) and Crohn’s disease (*Blakeley-Ruiz et al., 2019*). However, metaproteomic approaches have not been previously used for investigating the microbial community and function of the human microbiome in allergic disease.

This study therefore aimed to investigate the microbial community composition of the infant gut to identify key proteins playing metabolic functional roles in the microbiome of healthy infants and infants with atopic dermatitis in a Thai population-based birth cohort using a metaproteomic approach. Metaproteomic data were initially obtained from the gut microbiome of Thai infants by protein extraction from stool samples followed by LC-MS/MS-based protein identification. Thereafter, the data were processed with different bioinformatics tools and databases for protein quantitation and annotation. The obtained proteins were further classified into functional categories. To identify proteins that were uniquely expressed in healthy infants or infants with atopic dermatitis, the significant differentially expressed proteins (DEPs), metabolic functional roles, functional categories and taxonomic differences between healthy infants and infants with atopic dermatitis were then considered. To further identify reporter proteins associated with metabolic alterations between the healthy and disease groups, a PPI network was constructed and then integrated with a list of DEPs identified between the healthy and atopic dermatitis groups.

This study serves as a scaffold for microbial community-wide metabolic functional studies of the infant gut microbiome in relation to allergic disease. Our work provides the first metaproteomic data from a population-based birth cohort in a developing Southeast Asian country.

**Table 1:** Thai infant fecal sample features.

**Subject ID**	**H** **01**	**H** **02**	**H** **03**	**H** **04**	**H** **05**	**H** **06**	**H** **07**	**H** **08**	**H** **09**	**H** **10**	**H** **11**	**AD** **01**	**AD** **02**	**AD** **03**	**AD** **04**	**AD** **05**	**AD** **06**	**AD** **07**
Gestational age (week)	38	39	39	39	40	39	38	38	37	38	40	39	38	38	38	37	38	38
Gender	F	F	M	M	M	M	F	M	M	M	F	M	F	F	F	M	M	M
Age (months)	12	9	12	9	9	12	9	12	12	12	12	9	9	12	12	9	12	9
Delivery Mode	V	CS	V	V	V	CS	CS	CS	CS	V	V	V	CS	V	V	V	CS	V
Feeding Mode	Mix	Mix	Mix	Mix	Mix	Mix	Mix	Mix	Mix	Mix	Mix	Mix	Mix	Mix	Mix	Mix	Mix	Mix

**Notes.**

Group: H, healthy; AD, atopic dermatitis; Gender: M, male; F, female; Delivery mode: V, vaginal, CS, Cesarean-section; Feeding Mode: Mix, breastfed and formula fed.

## Materials and Methods

### Fecal sample collection and preparation

Fecal samples were collected from 18 infants who participated in a population-based allergy birth cohort study at King Chulalongkorn Memorial Hospital, Bangkok, Thailand. These infants included 11 healthy controls and 7 patients with atopic dermatitis. Atopic dermatitis was diagnosed by a pediatric allergist according to the criteria of the American Academy of Dermatology (*Eichenfield et al., 2017*). The study was approved by the Ethics Committee of King Chulalongkorn Memorial Hospital, Bangkok, Thailand, under approval reference number 358/58. Written informed consent was obtained from the parents or guardians of the participants. Information on gestational age, sex, age (months), the mode of delivery, and the type of infant feeding (breastfed and formula fed) are presented in Table 1. The stool samples were collected from 9–12 months of age and frozen at −80 °C until analysis. Sample preparation was conducted as previously described by Losuwannarak et al. (2019)). Briefly, frozen fecal samples were reconstituted in 50 mM phosphate buffer pH 7.0 and then vortexed well. After centrifugation for 10 min at 12,000 rpm to remove debris and some large particles (*Chassaing et al., 2012*), the solubilized protein remaining in the clear supernatant was collected. Total soluble protein was measured with a Lowry assay (File S1) using bovine serum albumin as a standard (*Lowry et al., 1951*). In 5 µg protein samples, disulfide bonds were reduced using 5 mM dithiothreitol in 10 mM ammonium bicarbonate at 60 °C for 1 h, followed by the alkylation of sulfhydryl groups by 15 mM *iodoacetamide* in 10 mM ammonium bicarbonate for 45 min in the dark at room temperature. For digestion, the protein samples were mixed with sequencing-grade trypsin (ratio of 1:20) (Promega, Germany) and incubated at 37 °C overnight. Prior to liquid chromatography-tandem mass spectrometry (LC-MS/MS) analysis, the digested protein (tryptic peptide) samples were dried and protonated with 0.1% formic acid before injection into the LC-MS/MS system.

### Liquid chromatography-tandem mass spectrometry

LC-MS/MS was *conducted* as previously described in Losuwannarak et al. (2019). Specifically, the tryptic peptide samples (100 ng) were injected in triplicates into an Ultimate™ 3000 Nano/Capillary LC System (Thermo Scientific) coupled to a Hybrid quadrupole Q-TOF impact II™ (Bruker Daltonics) equipped with a Nano-captive spray ionization (CSI) source. Here, peptides were enriched on a µ-Precolumn 300 µm i.d. X five mm C18 PepMap™ 100, 5 µm, 100 Å  (Thermo Scientific) and separated on a 75 µm I.D. × 15 cm and packed with Acclaim™ PepMap™ RSLC C18, 2 µm, 100 Å, nanoViper (Thermo Scientific). A mobile phase of solvent X (0.1% formic acid) and solvent Y (80% acetonitrile and 0.1% formic acid) were applied on the analytical column. A linear gradient of 5–55% solvent Y was used to elute the peptides at a constant flow rate of 0.30 µl/min for 30 min. Electrospray ionization was performed at 1.6 kV using the CaptiveSpray. Mass spectra (MS) and MS/MS spectra were achieved in the positive-ion mode over the range (m/z) 150–2,200 (Compass 1.9 software, Bruker Daltonics).

### Quantification and identification of proteins using bioinformatics tools and databases

For the quantification of proteins, MaxQuant (version 1.6.6.0) was used to quantify individual samples, and their MS/MS spectra were matched to the UniProt bacterial database by using the Andromeda search engine (*Tyanova, Temu & Cox, 2016a*). Label-free quantitation with MaxQuant settings was performed, which included (1) a maximum of two missed cleavages, (2) mass tolerance of 0.6 Daltons for the main search, (3) trypsin as the digestion enzyme, (4) carbamidomethylation of cysteine residues as a fixed modification, and (5) oxidation of methionine and acetylation of the protein N-terminus as variable modifications. Notably, peptides with a minimum of 7 amino acids and at least one unique peptide were required for protein identification. The protein false discovery rate (FDR) was set at 1% and estimated from the reverse searches of sequences. The maximal number of modifications per peptide was set to 5. For searches in FASTA files, a protein database of 10 candidate bacterial families selected from earlier reports of gut microbiome data from Thailand (*Kisuse et al., 2018; La-ongkham et al., 2015*), which included *Bacteroidaceae*, *Bifidobacteriaceae Enterococcaceae, Enterobacteriaceae, Erysipelotrichaceae, Lachnospiraceae, Lactobacillaceae, Prevotellaceae, Streptococcaceae* and *Veillonellaceae*, was downloaded from UniProt. Database with potential contaminants included in MaxQuant was automatically added. The MaxQuant ProteinGroups.txt file was subsequently obtained in conjunction with the use of Perseus software (version 1.6.6.0) for importing peptide sequences into the metaproteome dataset (Tyanova et al., 2016b). Exact peptides for which a unique protein sequence was matched to a single bacterial strain were classified as bacterial strain-specific sequences for taxonomic classification (Heyer et al., 2017; Karlsson et al., 2012). The remaining peptides for which a unique protein sequence was not matched to a single bacterial strain were discarded. The protein sequences assigned with protein IDs with known/putative functions from the UniProt bacterial database were denoted as annotated proteins. In contrast, the protein sequences assigned an ID corresponding to a hypothetical protein/uncharacterized protein were designated as unannotated proteins. Maximum peptide intensities were log_2_ transformed in Microsoft Excel, providing the protein expression levels (PELs) for DEPs analysis. The raw MS/MS spectra data, protein sequences, and PELs of the healthy and atopic dermatitis groups and the outlier observations from all samples were deposited in the Figshare data repository (figshare.com/s/1f8040674ef62c6d610c).

### Comparative microbial community composition and differentially expressed proteins analysis

To compare the microbial community composition according to the 10 selected bacterial families between the healthy and atopic dermatitis groups, the relative abundance of the microbial families was plotted using the ggplot2 package (*Wickham, 2009*) in R (version 3.5.3) (http://www.R-project.org). The Wilcoxon rank-sum test and multiple testing via false discovery rate (FDR) correction were performed to identify statistically significant differences (adjusted *p*-value < 0.05) in 10 selected bacterial families between the healthy and atopic dermatitis groups. Notably, the Wilcoxon rank-sum test was selected in this study because it is a nonparametric test that compare medians between two groups of independent samples. To identify targets of interest of bacterial families, an additional hierarchical clustering using the complete linkage method with Euclidean distance by the hclust function in the R stats package (version 3.5.3) (R Core Team, 2018) was applied to group similar patterns across 10 selected bacterial families in the healthy and atopic dermatitis groups. A *Z*-score >0.5 was set as a threshold, which was calculated by the total protein expression levels at the bacterial family level for selecting targets of interest among bacterial families (Yang et al., 2015). For further statistical analysis, Fisher’s exact test and FDR correction were also used for the analysis of each type of categorical data, such as subfunctional categories from the KEGG database (Kanehisa et al., 2004), across bacterial families between the healthy and atopic dermatitis groups.

In the DEPs analysis between the healthy and atopic dermatitis groups, the Wilcoxon rank-sum *test* and FDR correction were also used to identify significant proteins (adjusted *p*-value < 0.05). For the metabolic functional annotation of DEPs, the KEGG database was also used. To identify proteins playing metabolic functional roles that were uniquely expressed in the healthy infants or the infants with atopic dermatitis, a list of the significant proteins obtained from the DEPs analysis between the healthy and atopic dermatitis groups was then searched by using the jvenn viewer (Bardou et al., 2014).

### Identification of reporter proteins through protein–protein interaction network construction

Reporter feature analysis (Oliveira, Patil & Nielsen, 2008) was applied to identify reporter proteins. This is a hypothesis-driven method for the analysis of omics data. It combines the topology of the PPI network with the DEPs data between the healthy and atopic dermatitis groups and allows the identification of the reporter proteins around which DEPs changes are significantly concentrated. The applied reporter features algorithm was based on the constructed PPI network for the microbiome. To explore the PPI network constructed for the microbiome, the PPIs of *Escherichia coli* K12 extracted from the BioGRID database were used as a template and searched against all possible proteins identified from metaproteomic data using BLASTP under the assumption of the best match of protein homologs and interologs of microbiome shared with the *E. coli* K12 interaction set (*Jonsson et al., 2006*). To visualize the PPI network constructed for the microbiome, Cytoscape (version 3.7.2) was then used.

## Results

### Assessment of metaproteomic data from healthy Thai infants and Thai infants with atopic dermatitis

The metaproteomic data from the 18 Thai infant gut microbiomes resulted in the identification of 142,665 independent mass spectral counts (i.e., 85,430 spectral counts for 11 healthy samples and 57,235 spectral counts for 7 atopic dermatitis samples). After division to the number of samples in each group, the spectral counts per sample are presented between the healthy (7,767 spectral counts) and atopic dermatitis (8,177 spectral counts) groups in Table 2. Based on the Wilcoxon rank-sum *test*, the spectral counts per sample were comparable between the healthy and atopic dermatitis groups (File S2). According to all of the spectral counts assigned to the protein sequences on the basis of the spectral library and proteomic resources (*Li et al., 2013*), a greater number of total protein sequences with annotations were identified in the healthy samples (38,237 sequences) than in the atopic dermatitis samples (27,980 sequences). After considering the unique protein sequences, 49,973 annotated proteins out of 68,232 total proteins were selected for further analysis. To explore the taxonomy, functions, and pathways of the gut microbiome between the healthy and atopic dermatitis groups, a well-characterized *cohort* of Thai infants with ages of 9–12 months exhibiting the same feeding mode (breastfed or formula fed) and delivery mode was initially considered, as shown in Table 1. The results are described in the following section.

**Table 2:** Assigned spectral counts and unique protein sequences.

**Source**	**Protein annotation**	**Total spectral counts**^b^ **(Spectral counts per sample)**			**Total protein sequences**^b^		**Unique protein sequences**
		**H**	**AD**		**H**	**AD**	
Bacteria	Annotated proteins^1^	62,302 (5,664)	41,725 (5,961)		38,237	27,980	49,973
	Unannotated proteins	23,128 (2,103)	15,510 (2,216)		14,080	10,273	18,259
	Total	85,430 (7,767)	57,235 (8,177)		52,317	38,253	68,232

**Notes.**

H, healthy; AD, atopic dermatitis.

a Annotated protein is based on a protein ID with assigned function from Uniprot database.

b Spectral counts are the number of spectra assigned to protein sequence.

### Identification of microbial community composition profiles between healthy Thai infants and Thai infants with atopic dermatitis

**Figure 1:**
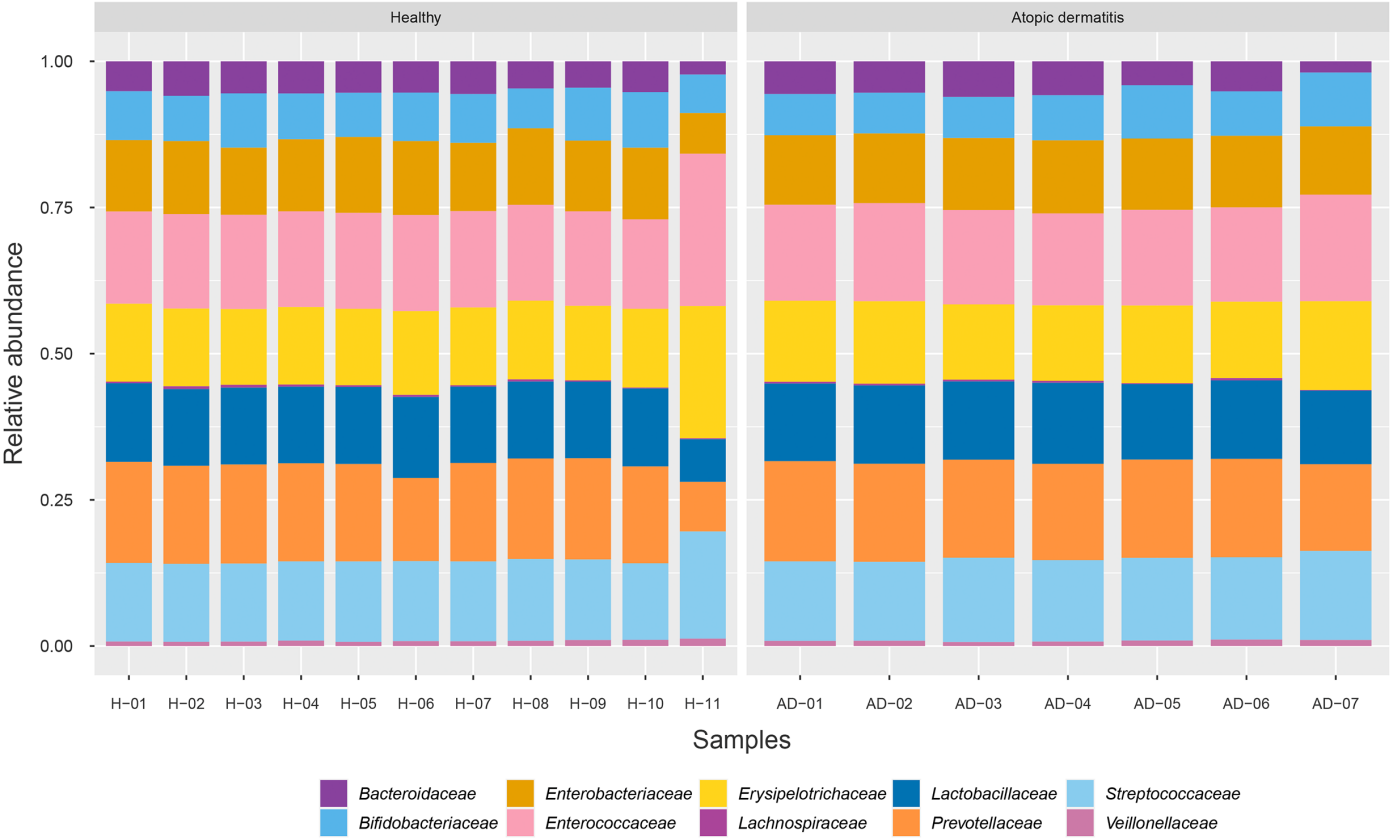
Relative abundance of 10 bacterial families in the healthy and atopic dermatitis groups. Each bar represents the average relative abundance of each bacterial family. Abbreviations: H, healthy; AD, atopic dermatitis.

**Figure 2:**
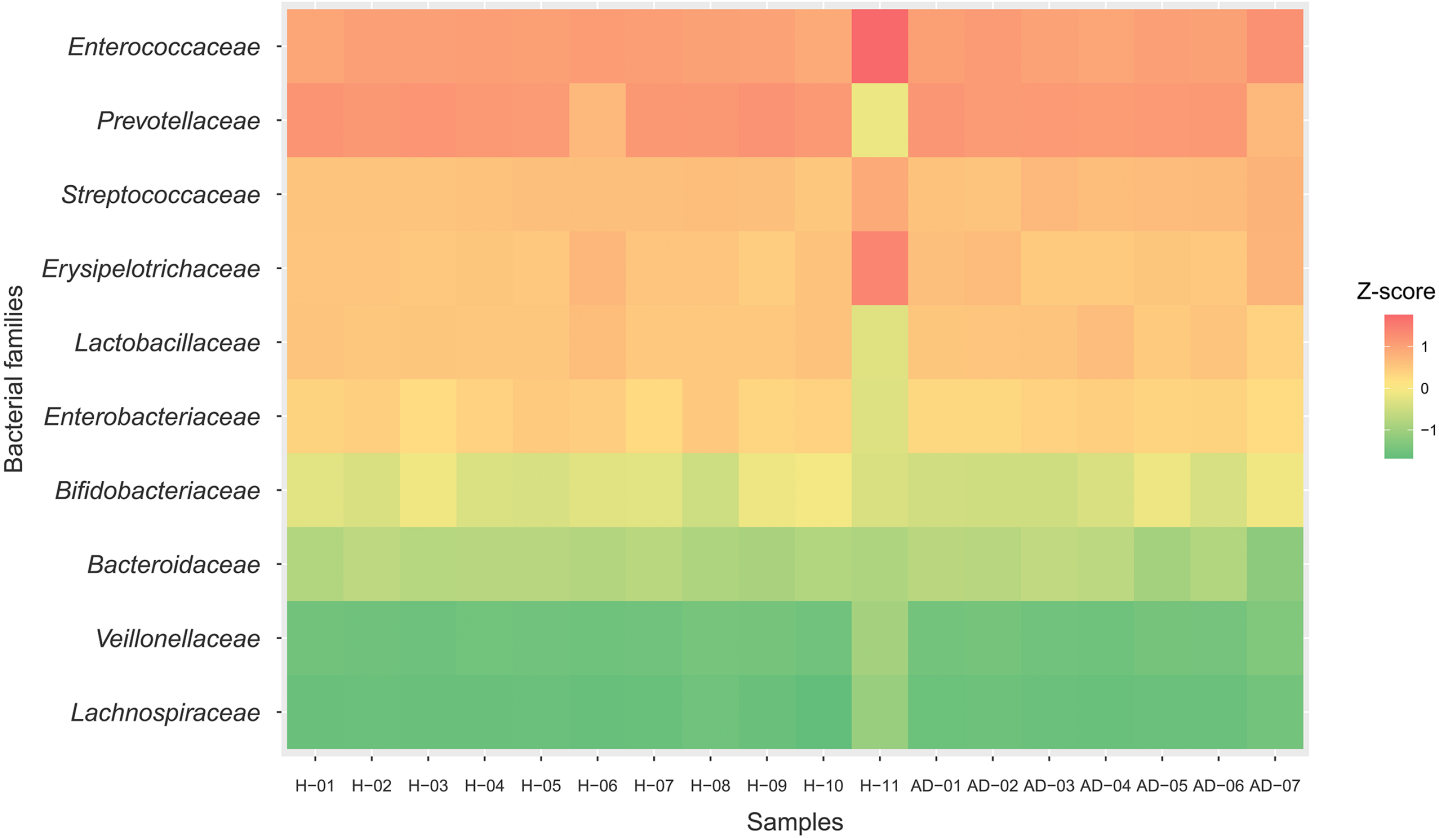
Relative protein expression of 10 bacterial families in the healthy and atopic dermatitis groups. A heatmap was generated by using the ggplot2 package (*Wickham, 2009*) implemented in (R Core Team, 2019) for visualization. Abbreviations: H, healthy; AD, atopic dermatitis.

To identify the microbial community composition profiles between the healthy and atopic dermatitis groups, a total of 68,232 proteins from the assessed metaproteomic data were analyzed at the bacterial family level. The relative abundance of the 10 selected bacterial families (see Materials and Methods) in the microbial community composition profiles was identified as shown in Fig. 1. The results were comparable between the healthy and atopic dermatitis groups across the 10 bacterial families. Using the Wilcoxon rank-sum test and FDR correction, we found non-significant differences in the bacterial families between the healthy and atopic dermatitis groups. This result shows that the patterns of the bacterial community composition in the infants were similar between the healthy and atopic dermatitis groups of our study at the ages of 9–12 months. Considering the high relative protein expression (*Z*-score > 0.5) observed in the majority of the samples from the healthy and atopic dermatitis groups across these 10 bacterial families, we interestingly found that the top five families were the same in healthy and atopic dermatitis groups, including *Enterococcaceae*, *Prevotellaceae, Streptococcaceae, Erysipelotrichaceae,* and *Lactobacillaceae,* as illustrated in Fig. 2 and Files S3–S5.

### Assignment of metabolic functions of the gut microbiome between healthy Thai infants and Thai infants with atopic dermatitis

**Figure 3:**
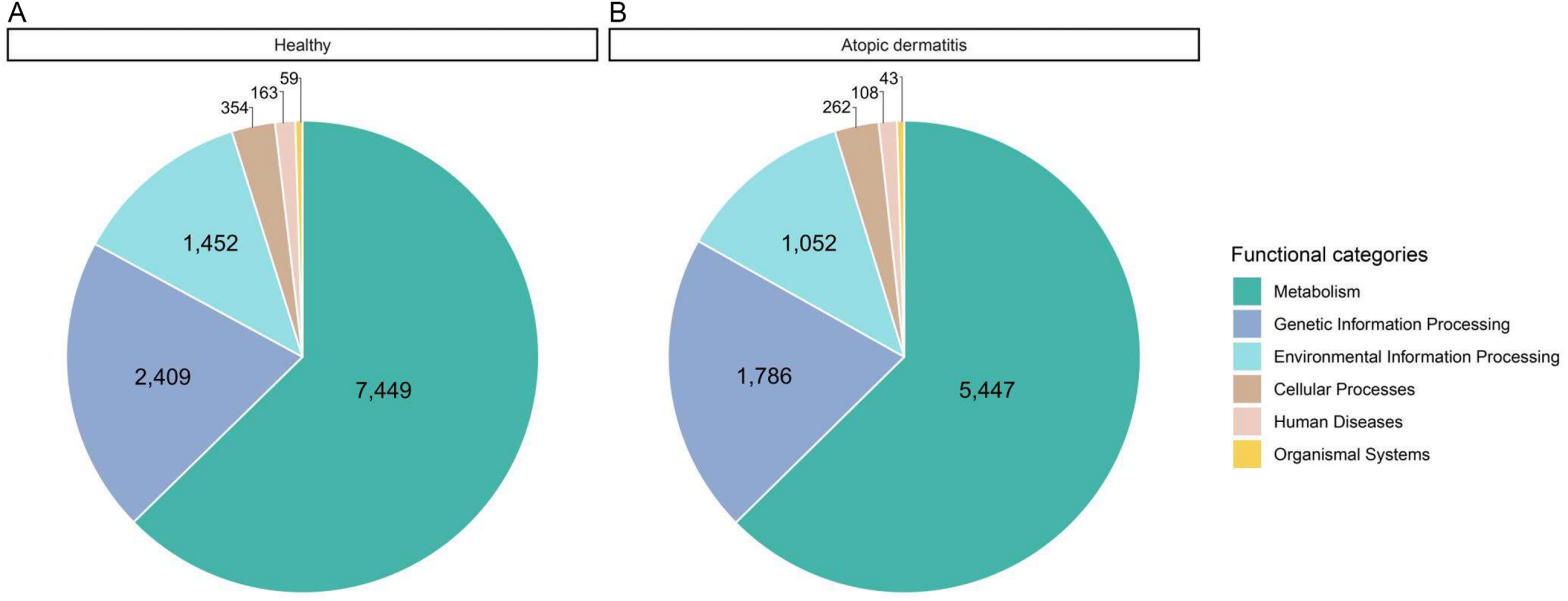
Comparative distribution of the main functional categories of metaproteome data between the (A) healthy and (B) atopic dermatitis groups based on the KEGG database. .

All possible proteins (i.e., 49,973 annotated proteins out of 68,232 total proteins from the assessed metaproteomic data) were functionally assigned according to the KEGG database (*Kanehisa et al., 2004*). Six functional categories (i.e., metabolism, genetic information processing, environmental information processing, cellular processes, human diseases and organismal systems) were classified as shown in Fig. 3 and File S6. Metabolism was the largest functional category among these categories. The number of proteins with assigned metabolic functions included in the metabolism category was higher in the healthy samples (7,449 proteins) than in the atopic dermatitis samples (5,447 proteins). It is worth noting that a number of proteins assigned metabolic functions depended on the total accessible protein sequences from the healthy or atopic dermatitis samples (Table 2) available in the KEGG database as well as the number of samples in each group.

**Figure 4:**
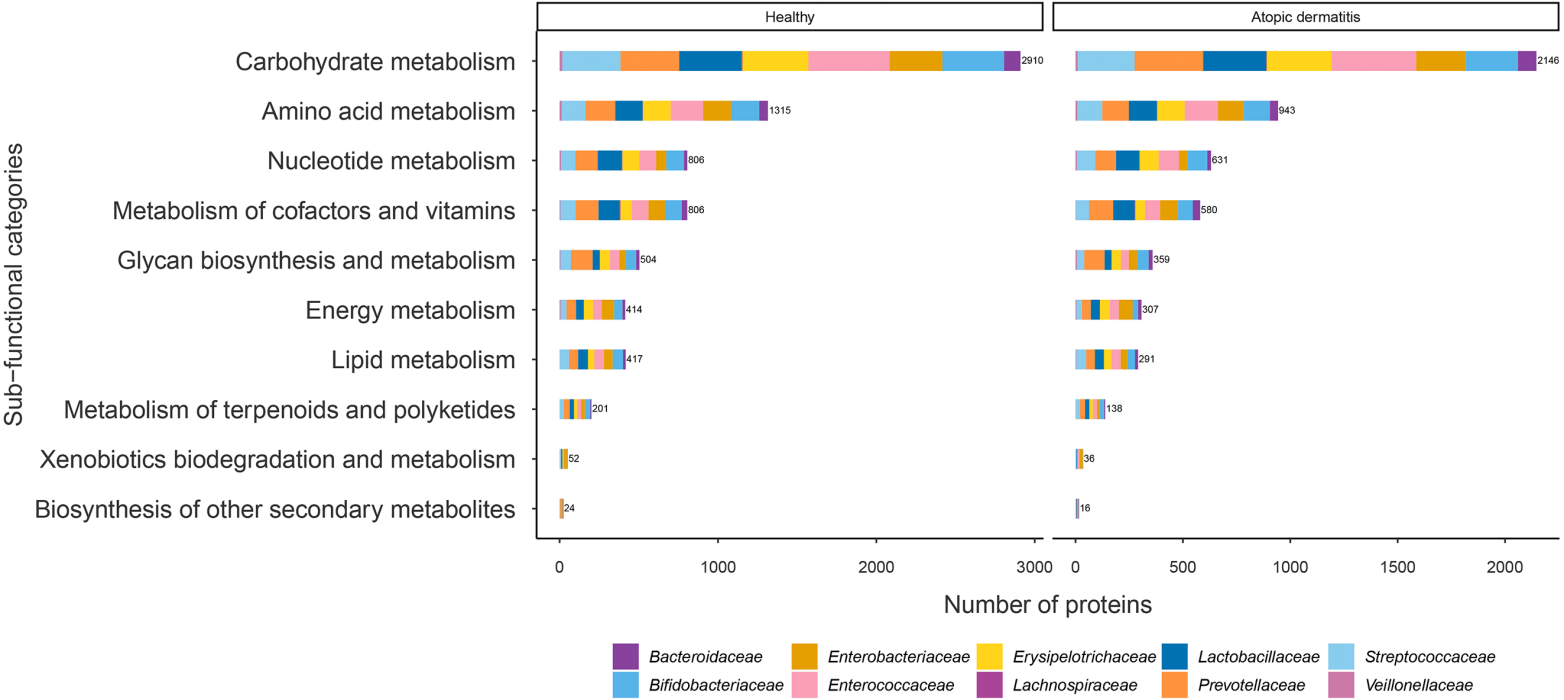
Comparison of the numbers of functionally assigned proteins between the healthy and atopic dermatitis groups across different metabolic functional categories based on the KEGG database. The horizontal bar chart shows the number of proteins devoted to different metabolic functional categories. Each color shows the group of proteins identified in each bacterial family level.

The proteins with assigned metabolic functions involved in the metabolism category were subcategorized, and the results are shown in Fig. 4 and File S7. Intriguingly, the numbers of proteins involved in carbohydrate metabolism (2,146 proteins), amino acid metabolism (943 proteins), nucleotide metabolism (631 proteins), the metabolism of cofactors and vitamins (580 proteins), glycan biosynthesis and metabolism (359 proteins), energy metabolism (307 proteins), lipid metabolism (291 proteins), the metabolism of terpenoids and polyketides (138 proteins), xenobiotic biodegradation and metabolism (36 proteins), and the biosynthesis of other secondary metabolites (16 proteins) were found to be lower in the atopic dermatitis group than in the healthy group (Fig. 4). The greatest numbers of proteins were related to carbohydrate metabolism in both the healthy and atopic dermatitis groups. After the application of Fisher’s exact test and FDR correction across the bacterial families in each subfunctional category, the results showed a non-significant difference in protein numbers between the healthy and atopic dermatitis groups (File S8). Further DEPs analysis is described in the next section.

### Analysis of differentially expressed proteins of the gut microbiome between the healthy and atopic dermatitis groups of Thai infants

A total of 49,973 annotated proteins from the assessed metaproteomic data were included in the DEPs analysis with the Wilcoxon rank-sum test and FDR correction. Focusing on the list of DEPs, 130 significant proteins were identified under an adjusted *p*-value <0.05 (File S9). Among these proteins, we observed two proteins of interest involved in metabolism: ornithine carbamoyltransferase (EC: 2.1.3.3), involved in arginine biosynthesis, from *Streptococcaceae*; and dihydroorotate dehydrogenase B (NAD^+^) (EC: 1.3.1.14), involved in pyrimidine metabolism, from *Lactobacillaceae*. The comparison of the PEL data revealed much higher PELs in the atopic dermatitis group than in the healthy group. These results suggest that the two identified proteins may be potential candidates for showing disease associations (*Ohnishi et al., 2017; Wozel, Vitéz & Pfeiffer, 2006*). To further identify proteins that were uniquely expressed in healthy infants or infants with atopic dermatitis, a list of the 130 significant proteins was searched by using the jvenn viewer (*Bardou et al., 2014*) (File S9). As expected, we found that 8 significant proteins were uniquely expressed in the majority of the samples from the atopic dermatitis group and showed no expression in all samples from the healthy group. Interestingly, these proteins were involved in metabolism, specifically carbohydrate metabolism (4 proteins), the metabolism of cofactors and vitamins (1 protein), amino acid metabolism (1 protein), nucleotide metabolism (1 protein), and xenobiotic biodegradation and metabolism (1 protein). The results are listed in Table 3. However, no proteins involved in metabolism were uniquely expressed in the majority of the samples from the healthy group while showing zero expression in all samples from the atopic dermatitis group.

**Table 3:** List of eight proteins uniquely expressed in atopic dermatitis samples.

**Protein IDs**	**Protein names**	**Bacteria**	**Families**	**Sub-functional categories**	**PELs^a^**
R6ZSA4	Demethylmenaquinone methyltransferase (EC: 2.1.1.163)	*Bacteroides* sp. CAG:714	*Bacteroidaceae*	Metabolism of cofactors and vitamins	13.16
A0A0H4QFW8	Asparagine synthase (glutamine-hydrolysing) (EC: 6.3.5.4)	*Lactobacillus ginsenosidimutans*	*Lactobacillaceae*	Amino acid metabolism	13.34
U1SDZ8	Triosephosphate isomerase (EC: 5.3.1.1)	*Alloscardovia omnicolens* F0580	*Bifidobacteriaceae*	Carbohydrate metabolism	15.73
A0A3D2W7G8	6-phospho-beta-glucosidase (EC: 3.2.1.86)	*Erysipelotrichaceae* bacterium	*Erysipelotrichaceae*	Carbohydrate metabolism	13.35
A0A3R6L4H6	Fructose-bisphosphate aldolase (EC: 4.1.2.13)	*Erysipelotrichaceae* bacterium AM17-60	*Erysipelotrichaceae*	Carbohydrate metabolism	14.24
A0A1H3YY32	Beta-xylosidase (EC: 3.2.1.37)	*Prevotella* sp. tc2-28	*Prevotellaceae*	Carbohydrate metabolism	13.53
A0A0F4KSW8	Uridine kinase (EC: 2.7.1.48)	*Lactobacillus mellis*	*Lactobacillaceae*	Nucleotide metabolism	13.47
A0A2A9IP29	Glyoxalase (EC: 4.4.1.5)	*Lactococcus lactis*	*Streptococcaceae*	Xenobiotics biodegradation and metabolism	14.37

**Notes.**

a Median values of PELs are presented.

Regarding the proteins involved in carbohydrate metabolism that were uniquely expressed in the samples from the atopic dermatitis group, very promisingly we identified triosephosphate isomerase (TPI) (PEL of 15.73, EC: 5.3.1.1) in *Alloscardovia omnicolens* F0580 from *Bifidobacteriaceae*. As shown in Table 3, we observed that other proteins that were uniquely expressed in samples from the atopic dermatitis group were involved in carbohydrate metabolism, such as 6-phospho-beta-glucosidase (PEL of 13.35, EC: 3.2.1.86) in *Erysipelotrichaceae* bacterium, fructose-bisphosphate aldolase (PEL of 14.24, EC: 4.1.2.13) in *Erysipelotrichaceae* bacterium AM17-60 from *Erysipelotrichaceae*, and beta-xylosidase (PEL of 13.53, EC: 3.2.1.37) in *Prevotella* sp. tc2-28 from *Prevotellaceae*.

Considering the metabolism of cofactors and vitamins, we very intriguingly identified demethylmenaquinone methyltransferase (DMM) as protein that was uniquely expressed (PEL of 13.16, EC: 2.1.1.163) in samples from the atopic dermatitis group identified in *Bacteroides* sp. CAG: 714. For the other remaining functional categories, the proteins that were uniquely expressed in samples from the atopic dermatitis group included asparagine synthase (glutamine hydrolysis) (PEL of 13.34, EC: 6.3.5.4) in *Lactobacillus ginsenosidimutans*, uridine kinase (PEL of 13.47, EC: 2.7.1.48) in *Lactobacillus mellis* from *Lactobacillaceae*, and glyoxalase (PEL of 14.37, EC: 4.4.1.5) in *Lactococcus lactis* from *Streptococcaceae*. As mentioned above, these identified microbial communities were shown to be potential components of the infant gut microbiome that could play metabolic functional roles with a profound effect on human health/disease in our cohort studies.

### Identification of reporter proteins underlying metabolic alterations between the healthy and atopic dermatitis groups of Thai infants

**Figure 5:**
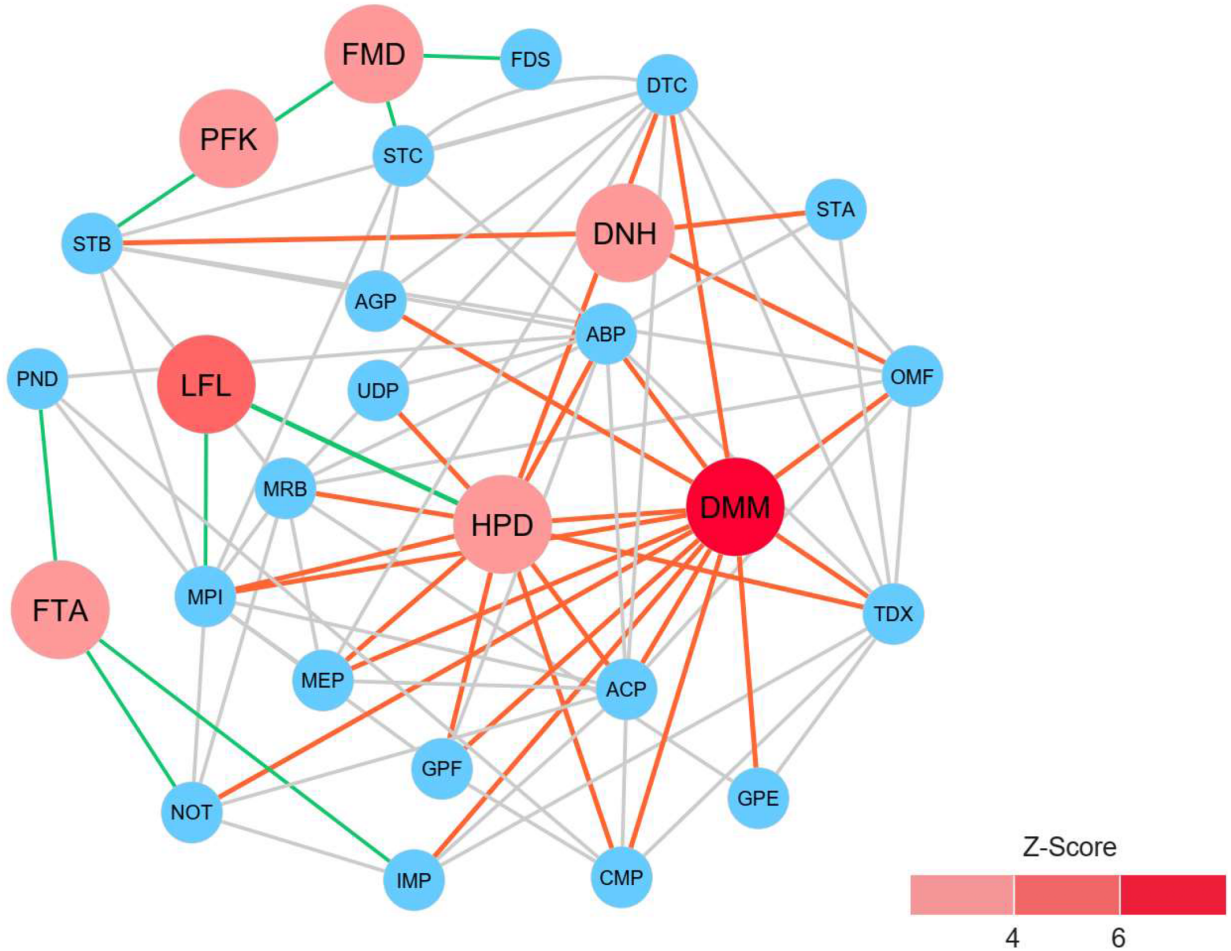
Reporter protein subnetwork involving ubiquinone and other quinone biosynthesis as well as energy supply. Node and edge represent protein and its interaction visualized by Cytoscape (version 3.7.2). A circle with red color represents reporter protein. A circle with blue color represents neighboring protein. A red edge means interactions between reporter proteins involved in ubiquinone and other quinone biosynthesis and other neighboring proteins. A green edge means interactions between reporter proteins involved in energy supply and other neighboring proteins. A grey edge means interactions between neighboring proteins.

To identify reporter proteins underlying the metabolic alterations between the healthy and atopic dermatitis groups, the reporter features algorithm was applied. Here, the reporter features algorithm was based on the constructed PPI network for the microbiome, integrated with a list the DEPs between the healthy and atopic dermatitis groups (see Materials and Methods). The *E. coli* K12 PPI template (4,062 proteins and 184,017 interactions) across all possible protein lists (49,973 annotated proteins) from the assessed metaproteomic data was searched, and a PPI network involving 3,995 proteins with 77,391 interactions was then constructed. After applying the reporter features algorithm with specific thresholds (*Z*-score ≥ 3.00), we identified the top 15 reporter proteins through PPI network analysis (File S10). Among these proteins, 7 reporter proteins were mainly related to metabolic functions of interest. As illustrated in Fig. 5, DMM was the most significant protein (*Z*-score of 6.931) in the subnetwork. This result clearly suggests that DMM potentially plays an important role in the metabolic alterations between the healthy and atopic dermatitis groups. Other reporter proteins were also identified in the subnetwork, which were associated with DMM in the biosynthesis pathways of ubiquinone and other quinones, such as 1,4-dihydroxy-2-naphthoyl-CoA hydrolase (EC: 3.1.2.28) and 4-hydroxy-3-polyprenylbenzoate decarboxylase (EC: 4.1.1.98). Moreover, we identified reporter proteins involved in the energy supply in the subnetwork (e.g., related to NADH and ATP production), such as F-type H^+^-transporting ATPase subunit delta (EC: 7.1.2.2), 6-phosphofructokinase (EC: 2.7.1.11), formate dehydrogenase subunit gamma (EC: 1.17.1.9), and long-chain-fatty-acid-[acyl-carrier-protein] ligase (EC: 6.2.1.20). Accordingly, the biosynthesis of ubiquinone and other quinones as well as the energy supply to the gut bacteria might influence host physiology and health.

## Discussion

This study shows the prospective benefits of metaproteomics-based analysis in research on the human gut microbiome. Based on our metaproteomics analysis, we revealed the complex nature of the microbiome taxa by identifying microbial community composition profiles and related key proteins involved in the metabolic functions of healthy Thai infants and Thai infants with atopic dermatitis. In the direct comparison of the taxonomy and high relative protein expression between the healthy and atopic dermatitis groups across 10 selected bacterial families (Figs. 1 and 2), the results clearly identified five families abounded in both the healthy and atopic dermatitis groups, including *Enterococcaceae*, *Prevotellaceae, Streptococcaceae, Erysipelotrichaceae, and Lactobacillaceae*. Considering the similar characteristics of the subjects in the cohort e.g., showing the same feeding patterns (Table 1), these five bacterial families might present critical activities involved in host physiology and/or host disease. In addition to the taxonomic information provided through metaproteomics analysis, a quantitative overview was generated at the metabolic function level. In DEPs analysis, the identification of significant proteins involved in metabolism that were uniquely expressed in samples from the atopic dermatitis group was of particular interest. Among 8 significant proteins, very promisingly we found that TPI was identified in *A. omnicolens* F0580. Considering the observed high PEL (Table 3), TPI might be related to *allergic* reactions, as supported by Yang et al. (2017). In addition, *A. omnicolens* is a member of the *Bifidobacteriaceae* family and *Alloscardovia* genus. It normally inhabits the gastrointestinal tract of humans, and an earlier report indicated that *A. omnicolens* is associated with infectious disease (Mahlen & Clarridge, 2009). However, this species has been infrequently isolated from human clinical specimens because it is catalase and oxidase negative and has the morphology of short irregularly shaped rods (Mahlen & Clarridge, 2009). Unexpectedly, our metaproteomic study identified *A. omnicolens* F0580 together with TPI as being related to metabolic function among the gut bacterial community. Taken together, these results suggest that *A. omnicolens* F0580 might be considered a potential contributing pathogen (Mahlen & Clarridge, 2009). Of particular interest, considering the metabolism of cofactors and vitamins, we observed DMM as a protein that was uniquely expressed in samples from the atopic dermatitis group, which was identified in *Bacteroides* sp. CAG: 714. In general, *Bacteroides* sp. are inhabitants of the human gut presenting mutualistic benefits to humans because of their ability to prevent pathogens from colonizing the gut. However, when the relative abundance of *Bacteroidaceae* was reduced, low PELs were found in the human gut in both the healthy and atopic dermatitis groups, as supported by Figs. 1 and 2, respectively, making the gut a more favorable environment for pathogenic bacteria, potentially resulting in gut dysbiosis and secondary infection. Further concerning metabolic functions identified in samples from the atopic dermatitis group, DMM is involved in the final step of menaquinone biosynthesis, in which it catalyzes methylation of demethylmenaquinone using *S*-adenosylmethionine, resulting in the formation of menaquinone. Taken together, the results suggest that *Bacteroides* sp. CAG: 714 in the human gut could produce menaquinone, which may be an alternative source of vitamin K in patients (Ramotar et al., 1984). Some bacteria in the human gut can meet their energy needs through the use of menaquinone, which represents an essential point of vulnerability in the electron transport chain for the synthesis of adequate amounts of ATP (Sukheja et al., 2017). This suggests that the relationship between menaquinone and the gut microbiota (Karl et al., 2015) might play important roles in the mechanism of atopic dermatitis. Following the identification of reporter proteins underlying metabolic alterations between the healthy and atopic dermatitis groups, 7 reporter proteins were found to show metabolic alterations between the healthy and disease groups. Notably, DMM was shown to be the most significant protein. This result clearly supports a potential important role of DMM in metabolic alterations between the healthy and atopic dermatitis groups. In addition, we found that other reporter proteins were involved in the energy supply, indicating that the energetic contribution of the gut bacteria might influence host physiology and health. An energetic imbalance between the gut microbiota and the host could be a possible risk factor for allergic diseases, such as atopic dermatitis.

The present study supports metaproteomics as a potentially valuable approach for use in routine medical diagnostics, such as human feces analysis. The success of metaproteomic studies of the human gut microbiome depends on different aspects of the experimental design and the available bioinformatics resources, such as the cohort design, sample sizes, spectral libraries, proteomic resources, and bioinformatics databases and tools. A great challenge in facilitating microbiome analysis is to integrate metaproteomics as a complementary approach to other meta-omics techniques (e.g., metagenomics) with the aim of achieving a comprehensive understanding of human and microbiome interactions in relation to health and disease (*Morowitz et al., 2011; Sharon et al., 2013; Petriz & Franco, 2017*).

## Conclusions

Metaproteomics-based analysis reveals the taxonomy, function, and metabolic pathways of the gut microbiome. Through metaproteomics, we may monitor the gut microbiome and assess its impact on health and allergic disease.

##  Supplemental Information

10.7717/peerj.9988/supp-1 Supplemental Information 1 Analysis of the infant gut microbiome between healthy infants and infants with atopic dermatitis using metaproteomics

## Additional Information and Declarations

### Data Availability

The following information was supplied regarding data availability: Raw MS/MS spectra data are available in ProteomeXchange: PXD021324 and JPST000958, protein sequences, PELs of healthy and atopic dermatitis samples, and outlier observation of all samples are available at Figshare: Kingkaw, Amornthep (2020): Analysis of the infant gut microbiome reveals metabolic functional roles associated with healthy infants and infants with atopic dermatitis using metaproteomics. figshare. Dataset. https://doi.org/10.6084/m9.figshare.12154560.v2.
